# A *de Novo* ZMIZ1 Pathogenic Variant for Neurodevelopmental Disorder With Dysmorphic Facies and Distal Skeletal Anomalies

**DOI:** 10.3389/fgene.2022.840577

**Published:** 2022-03-31

**Authors:** Guanting Lu, Liya Ma, Pei Xu, Binqiang Xian, Lianying Wu, Jianying Ding, Xiaoyan He, Huiyun Xia, Wuwu Ding, Zhirong Yang, Qiongling Peng

**Affiliations:** ^1^ Deyang Key Laboratory of Tumor Molecular Research, Department of Pathology, Translational Medicine Research Center, Deyang People’s Hospital, Deyang, China; ^2^ Department of Child Healthcare, Shenzhen Baoan Women’s and Children’s Hospital, Jinan University, Shenzhen, China

**Keywords:** Zmiz1, NEDDFSA, Chinese, low-complexity region, whole-exome sequencing

## Abstract

**Background:** Neurodevelopmental disorder with dysmorphic facies and distal skeletal anomalies (NEDDFSA) is a rare syndromic disorder characterized by global neurodevelopmental delay, early-onset hypotonia, poor overall growth, poor speech/language ability, and additional common phenotypes such as eye anomalies, joint hypermobility, and skeletal anomalies of the hands and feet. NEDDFSA is caused by heterozygous pathogenic variants in the *ZMIZ1* gene on chromosome 10q22.3 with autosomal dominant (AD) mode of inheritance. All the 32 reported cases with variants in *ZMIZ1* gene had a genetic background in Caucasian, Hispanic, North African, and Southeastern Asian. Until now, there are no reports of Chinese patients with *ZMIZ1* pathogenic variants.

**Methods:** A 5-year-old girl was found to have the characteristic phenotypes of NEDDFSA. Array-Comparative Genomic Hybridization (array-CGH) and whole exome sequencing (WES) were applied for the trio of this female patient. Sanger sequencing was used to verify the selected variants. A comprehensive molecular analysis was carried out by protein structure prediction, evolutionary conservation, motif scanning, tissue-specific expression, and protein interaction network to elucidate pathogenicity of the identified ZMIZ1 variants.

**Results:** The karyotype was 46, XX with no micro-chromosomal abnormalities identified by array-CGH. There were 20 variants detected in the female patient by WES. A *de novo* heterozygous missense variant (c.2330G > A, p.Gly777Glu, G777E) was identified in the exon 20 of *ZMIZ1*. No variants of *ZMIZ1* were identified in the non-consanguineous parents and her healthy elder sister. It was predicted that G777E was pathogenic and detrimental to the spatial conformation of the MIZ/SP-RING zinc finger domain of ZMIZ1.

**Conclusion:** Thus far, only four scientific articles reported deleterious variants in *ZMIZ1* and most of the cases were from Western countries. This is the first report about a Chinese patient with *ZMIZ1* variant. It will broaden the current knowledge of ZMIZ1 variants and variable clinical presentations for clinicians and genetic counselors.

## Introduction

Neurodevelopmental disorder with dysmorphic facies and distal skeletal anomalies (NEDDFSA; OMIM #618659) is a rare syndromic disorder characterized by global neurodevelopmental delay, hypotonia, poor overall growth, poor speech/language ability, and other common phenotypes such as eye anomalies, joint hypermobility, and distal skeletal anomalies of the hands and feet (Carapito et al., 2019). A balanced translocation t (10; 19) (q22.3; q13.33) was first reported in 2015, involving zinc finger MIZ-type containing 1 (*ZMIZ1*, OMIM #607159) and proline-rich protein 12 (*PRR12*, OMIM #616633). It produced two types of fusion genes, ZMIZ1-PRR12 and PRR12-ZMIZ1, which might be related to the occurrence of intellectual disability (ID) and neuropsychiatric alterations (Córdova-Fletes et al., 2015). Later, in 2019, pathogenic variants involving the gene ZMIZ1 were identified in a cohort of 19 NEDDFSA cases from a transatlantic collaborative effort (Carapito et al., 2019). In the same year of 2019, an affected father and his two sons were identified to be suffering from the ZMIZ1-related neurodevelopmental disorder in Florida (Latchman et al., 2020). In 2021, Phetthong et al. reported a 5-year-old Thai girl with developmental delay, facial phenotypes resembling Williams syndrome, and cardiac defects. She carried three types of compound variants, a heterozygous *ZMIZ1* variant (c.1497+2T > C), a heterozygous frameshift variant of *OTUD6B* (OMIM #612021) (c.873delA, p.Lys291AsnfsTer3), and a 0.118 Mb 8q21.3 microdeletion involving *OTUD6B* (Phetthong et al., 2021).

The gene ZMIZ1 was mapped to chromosome 10q22.3 and it contains 21 exons to produce a 1067-amino acid protein with a calculated molecular mass of 123 kDa (Sharma et al., 2003). According to the Conserved Domain database (CDD) (Lu et al., 2020), ZMIZ1 contains a Zmiz1 N-terminal tetratricopeptide repeat domain (Zmiz1_N, 8–100), Med15 domain (184–557), a nuclear localization signal (NLS, 697–711), a SP-RING zine finger domain (SP-RING_ZMIZ, 739–786), and a transactivation domain (TAD, 837–1067). In 1999, Nagase et al. identified the gene *ZMIZ1* (previously called KIAA1224) from a fetal brain cDNA library (Nagase et al., 1999). The encoded protein is a transcriptional co-activator, which belongs to the Protein Inhibitor of Activated STAT (PIAS) family. As a member of the PIAS family, ZMIZ1 has a highly conserved MIZ (Msx-interacting zinc finger) domain which is important for protein-protein interaction and SUMOylation (Sharma et al., 2003; Beliakoff and Sun, 2006). It had been reported that ZMIZ1 could regulate the activity of many transcription factors, such as androgen receptor (AR) (Beliakoff and Sun, 2006), SMAD3 (Li et al., 2006), SMAD4 (Li et al., 2006), and p53 (Lee et al., 2007). As an ortholog of ZMIZ1, tonalli (tna) was identified in *Drosophila melanogaster* and interacted with the ATP-dependent SWI/SNF complexes, which suggested a potential role in chromatin remodeling (Gutiérrez et al., 2003). Recently, ZMIZ1 was identified to be interacted with BRG1 (SMARCA4) (Li et al., 2011), BAF57 (SMARCE1) (Li et al., 2011), or SATB1 (Pinnell et al., 2015) to regulate the chromatin remodeling in humans. Chromatin remodeling complex could regulate the expression of genes which were essential for the normal dendrite development, synaptic plasticity, and synapse formation (Wu et al., 2007; Vogel-Ciernia et al., 2013; Vogel-Ciernia and Wood, 2014). It has been reported that *in utero* electroporation of ZMIZ1 pathogenic variants into the progenitor cells in the ventricular zone (VZ) of mice cortices (E14.5) resulted in impaired neuronal positioning with an accumulation in the ventricular and subventricular zones (VZ/SVZ) and intermediate zone (IZ) and a corresponding depletion in the upper cortical plate (CP). Therefore, *ZMIZ1* variants were regarded as the causal genetic factors for NEDDFSA (Carapito et al., 2019).

Thus far, no patients with *ZMIZ1* variants have been reported in Chinese. In order to decipher the genetic factors for neurodevelopmental disorder or intellectual disability (NEDD/ID) in China, array-CGH and WES were carried out for a cohort of 54 patients with NEDD/ID living in Shenzhen, Guangdong Province, China. After comprehensive bioinformatic analysis, a *de novo* missense variant (c.2330G > A, p.Gly777Glu, or p.G777E) was identified in the exon 20 of *ZMIZ1* in a 5-year-old girl with mild development delay, mild intellectual disability, bilateral hip dysplasia, joint hypermobility, amblyopia in both eyes, strabismus in the right eye, and dysmorphic facial features. According to the criteria proposed by the American College of Medical Genetics and Genomics (ACMG) (Richards et al., 2015), this variant was classified as PS2 + PM1 + PM2 + PP2 + PP3 and annotated as “Pathogenic.” After comparing the clinical phenotypes described for NEDDFSA with the clinical phenotypes of our current Chinese patient, this girl was diagnosed as NEDDFSA. This variant is located in the highly conserved zf-MIZ domain and affected the three-dimensional conformation which might be detrimental for the binding of ZMIZ1 to its partners.

To our knowledge, this is the first case of Chinese NEDD/ID caused by a *ZIMI1* variant. Due to the huge population, more patients with ZMIZ1-related disorder will be found in the near future.

## Methods

### Sample Collection

This study was conducted in accordance with the Code of Ethics of the World Medical Association (Declaration of Helsinki) for experiments involving humans. This study was approved by the Ethics Committee of the Shenzhen Baoan Women’s and Children’s Hospital. Written informed consent was obtained from each individual.

Peripheral venous blood was collected from the 54 NEDD/ID patients and their parents. Genomic DNA was extracted using the TIANamp Blood DNA Kit (DP348, Tiangen Biotech, Beijing, China) according to the manufacturer’s instructions.

### Array-Comparative Genomic Hybridization

Array-CGH was performed using the Fetal DNA Chip (Version 1.2) designed by The Chinese University of Hong Kong (CUHK) (Leung et al., 2011; Huang et al., 2014). The chip contains a total of 60,000 probes for more than 100 diseases caused by known microduplication/microdeletions. It does not include small-size chromosomal abnormalities, copy number polymorphism, chimerism, or chromosomal rearrangement (Iafrate et al., 2004). The experimental procedures were carried out according to the standard Agilent protocol (Agilent Oligonucleotide Array-Based CGH for Genomic DNA Analysis, version 3.5). Hybridized slides were scanned with SureScan High-Resolution Microarray Scanner (G2505B, Agilent Technologies, Santa Clara, CA), and the image data were extracted and converted to text files using Agilent Feature Extraction software (Version 10.5.1.1). The data were graphed and analyzed using Agilent CGH Analytics software.

Only gains or losses that were encompassed by at least three consecutive oligomers on the array were considered. Then, the clinical relevance of observed chromosomal aberrations was estimated according to data found in the scientific literature and databases for each of the regions and genes involved, using the DECIPHER database (Swaminathan et al., 2012) for known microdeletion and microduplication syndromes and the Online Mendelian Inheritance in Man (OMIM) (Sayers et al., 2021) for known disease-causing genes, gene functions, and inheritance patterns. Copy number variations were considered as “likely pathogenic/pathogenic” when they involved regions known to be associated with microdeletion or microduplication syndromes.

### High-Throughput Whole Exome Sequencing

WES was performed for family trios (trio-WES) without chromosomal abnormalities at MyGenostics or BerryGenomics Co. LTD. Briefly, the fragmented genomic DNAs were ligated with the 3ʹ end of the Illumina adapters and amplified by polymerase chain reaction (PCR). The amplified DNA was captured with Gencap Human whole Exon Kit (52M) at MyGenostics or with xGen Exome Research Panel v2.0 (Integrated DNA Technologies, Coralville, IA) at BerryGenomics. The capture procedure was performed in accordance with the manufacturer’s protocol. Finally, the generated libraries were sequenced on Illumina HiSeq 2500 platform for paired-end sequencing.

**FIGURE 1:**
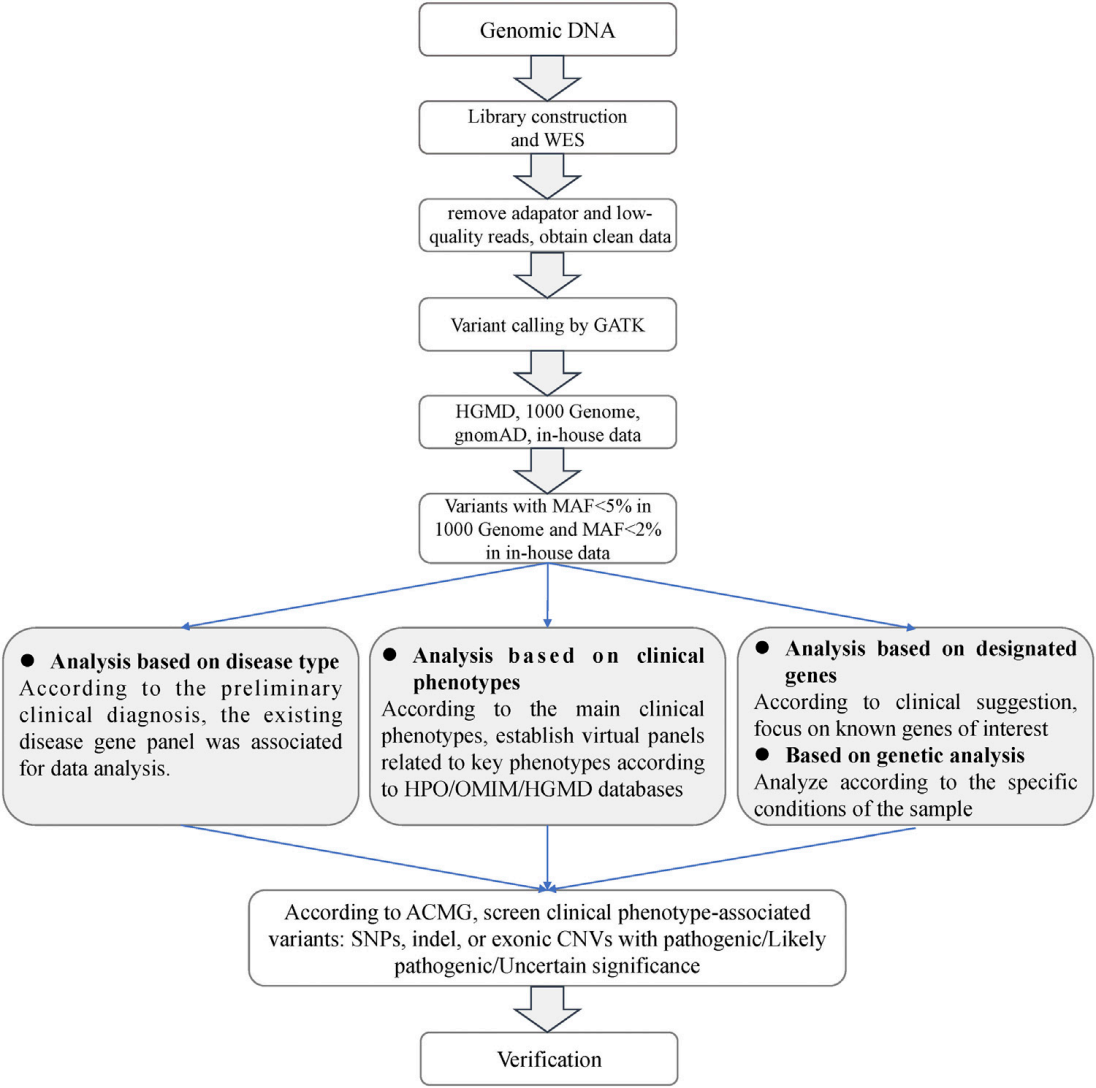
Analysis flowchart of the whole exome sequencing data.

The sequencing depth of each sample was about 100. Sequencing reads were aligned with the human reference genome (UCSC hg19). The workflow of the screening for causal variants was depicted in Figure 1. Briefly, clean reads were obtained after removal of adaptors and low-quality reads. GATK (Genome Analysis Toolkit) was used to trim the variant calling in the trimmed WES clean data (https://gatk.broadinstitute.org/hc/en-us). ANNOVAR was applied to annotate the generated VCF file (Wang et al., 2010). Deleted variants with a minor allele frequency (MAF) > 5% in the 1000 Genome Project, MAF >2% in in-house data, or synonymous single nucleotide variants (SNVs) were removed. SNVs that caused splicing, frameshift, stop gain, or stop loss were retained for subsequent analysis. A position was called as heterozygous if 25% or more of the reads identify the minor allele.

The chromosomal location and type of the identified variants were retrieved in UCSC Genome Browser (Navarro Gonzalez et al., 2021) and NCBI dbSNP (Bhagwat, 2010). The MAFs of the variants were screened in several public databases with a large number of human samples, such as 1000 Genome Project (*n* = 2504) (Siva, 2008), NHLBI Exome Sequencing Project (GO-ESP) (*n* = 6503) (Amendola et al., 2015), The Exome Aggregation Consortium (ExAC) (*n* = 60,706) (Lek et al., 2016), gnomAD (*n* = 15,708) (Scheps et al., 2020), and NHLBI Trans-Omics for Precision Medicine (TOPMED) (*n* = 60,000) (Taliun et al., 2021). The function prediction of these variants was carried out by online software, PolyPhen-2 (Adzhubei et al., 2010), and PROVEAN (Choi et al., 2012). Pathogenicity of the variants was evaluated according to the American College of Medical Genetics and Genomics (ACMG) guidelines (Richards et al., 2015). The selected variants were verified by Sanger sequencing using the ABI 3500 Genetic Analyzer (Applied Biosystems, Foster City, CA).

### Computational Analysis for the G777E Variant of ZMIZ1

Protein sequences of ZMIZ1 in 34 species were downloaded from NCBI GenBank, including five primates (*Homo sapiens*, *Pan troglodytes*, Gorilla *Gorilla gorilla*, *Hylobates moloch*, and *Macaca fascicularis*), one cattle (*Bos taurus*), one horse (*Equus caballus*), one dog (*Canis lupus* familiar), three carnivores (Neogale vison, *Panthera tigris*, and Halichoerus grypus), three rodents (*Eptesicus fuscus*, *Mus musculus*, and *Rattus norvegicus*), five reptiles (Crotalus tigris, *Varanus komodoensis*, Dermochelys coriacea, Chelonoidis abingdonii, and Mauremys mutica), two birds (*Falco rusticolus*, *Gallus gallus*), three amphibians (*Bufo bufo*, *Xenopus* tropicalis, and *Oryzias latipes*), three fish (*Takifugu rubripes*, Hippocampus comes, and *Danio rerio*), three arthropods (Limulus polyphemus, *Penaeus monodon*, and Ceratitis capitata), and four mollusks (Acropora millepora, *Crassostrea gigas*, Octopus sinensis, and Exaiptasia diaphana). The protein sequences were aligned by the ClustalW alignment algorithms of MEGA X (Kumar et al., 2018) (gap opening penalty and gap extension penalty for pairwise alignment and multiple alignment were set as 10.00, 0.10 and 10.00, 0.20, respectively; the delay divergent cutoff was 30%).

The intrinsically disordered regions of ZMIZ1 protein (NP_065071) were analyzed using the online web server IUPred2A (https://iupred2a.elte.hu/) with long disorder setting to identify probable disordered regions using the IUPred2 model and disordered binding regions using the ANCHOR2 model (Erdos and Dosztanyi, 2020). The distinct motifs of ZMIZ1 were analyzed using the online software Motif Scan (https://myhits.sib.swiss/cgi-bin/motif_scan) under default settings to search all known motifs in HAMAP (Pedruzzi et al., 2015), PROSITE (Hulo et al., 2006), Pfam (Mistry et al., 2021), and InterPro databases (Mitchell et al., 2019). The possible phosphorylation sites of ZMIZ1 were predicted by Disorder Enhanced Phosphorylation Predictor (DEEP, http://www.pondr.com/cgi-bin/depp.cgi) using 0.50 as the cutoff value (Iakoucheva et al., 2004).

The effect of G777E on the structural change was predicted by the online protein structure and function prediction tool, I-TASSER (Iterative Threading ASSEmbly Refinement) under default parameters (Yang and Zhang, 2015) for the whole second globular region (aa575-820) and visualized using Mol* 3D Viewer (Sehnal et al., 2021). The gene expression data of ZMIZ1 were evaluated according to the normalized signal intensity of probe 212124 at which were extracted from a gene atlas of human protein-encoding transcriptomes for 79 human tissues (NCBI GEO #GSE1133) (Su et al., 2004). The protein interaction network with ZMIZ1 (PPI enrichment *p* value = 1.51E-03) was generated by STRING (version 11.5, https://string-db.org/) under default settings. Gene Ontology (GO) analysis was performed on the nine members of the network in the GO knowledgebase (http://geneontology.org/) under default parameters.

## Results

### Sample Description

**FIGURE 2:**
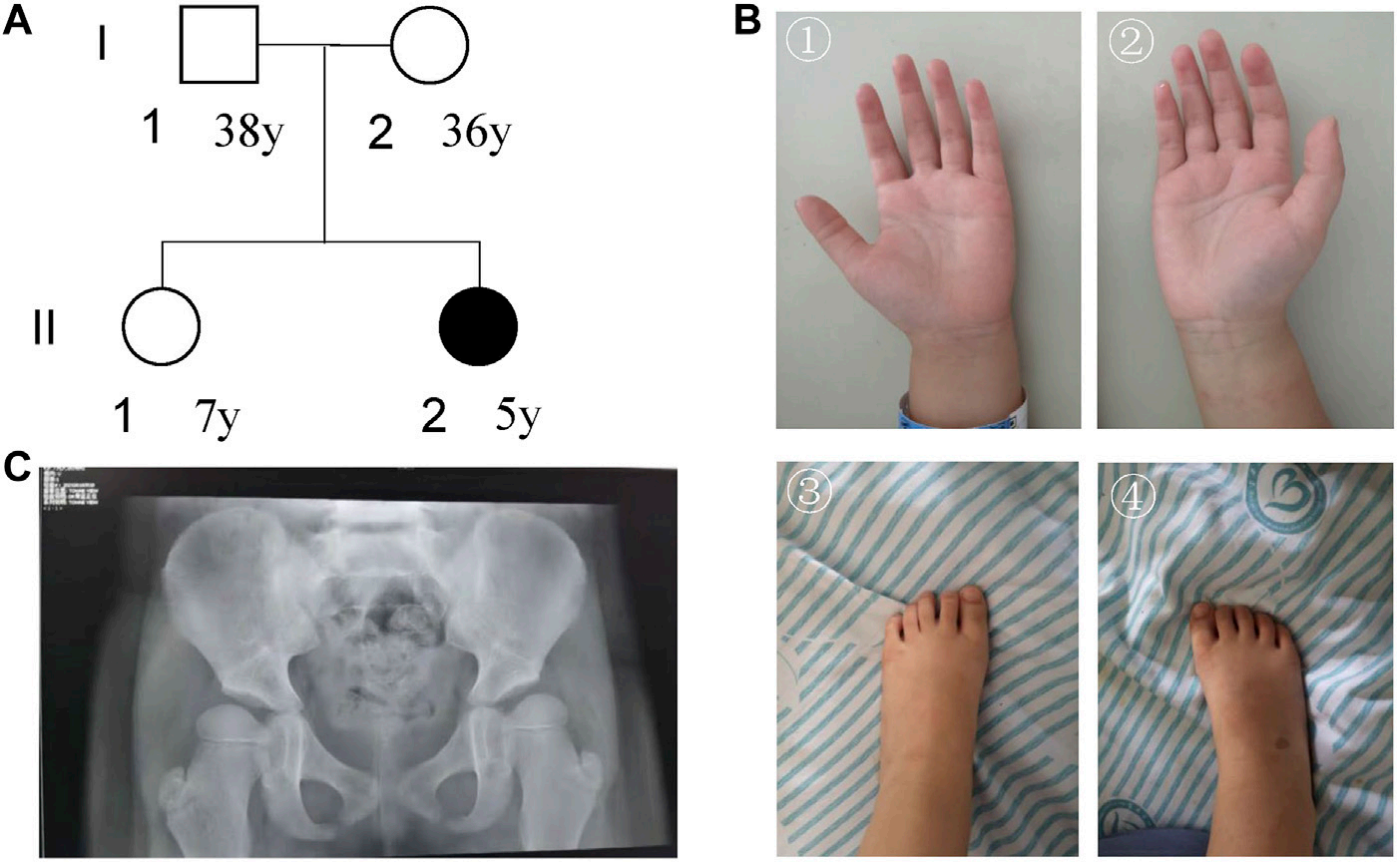
Characterization of the patient’s information. **(A)** Pedigree; **(B)** pictures of hands and foot; **(C)** DR X-ray film for bilateral hip joints.

There were 54 cases in our current NEDD/ID cohort collected from southern China. There were 48.15% (26/54) women and 51.85% (28/54) men. The mean age of women and men patients was 2.45 ± 1.15 and 2.67 ± 1.71, respectively. In these 54 samples, pathogenic variants were found in 33 patients, 5 with microdeletions and 28 with variants in protein-coding genes (Supplementary Table S1). The positive rate was 61.11% (33/54). In one patient, *ZMIZ1* was detected to have a pathogenic missense variant (c.2330G > A, p.G777E). This patient was a 5-year-old girl who was referred to our department because of psychomotor developmental delay. She was the second child of a non-consanguineous couple (Figure 2A). The proband was delivered at term to a 36-year-old mother by Cesarean section due to breech position at 2016-10. Her birth weight was 3000 g, and there was no history of asphyxia at birth. At the sixth month after birth, asymmetric dermatoglyphs were found on both of her lower limbs after a physical examination and later diagnosed as dysplasia of bilateral hip joints. At 1 year old, the patient had chronic constipation. Since 2017, she has been sent to the ophthalmology department several times due to binocular weak eyesight and strabismus in the right eye. After 1 year old, she was still unable to speak and walk without support and was sent to the rehabilitation center for special training. Until 2 years old, she was able to speak simple words and walk, and was finally diagnosed as “developmental delay”.

For **facial features**, the patient had epicanthus, ptosis, up-slanting eyelid fissure, wide eye distance, wide nose bridge, Cupid lip arch and low-set ear. Regarding **skeletal abnormalities**, she had short fingers and toes, conical fingers (Figure 2B), and excessive joint mobility. As for the **gross motor**, the patient could walk alone. She could not stand on one foot for more than 3 s and jump on one foot. The trotting posture was slightly abnormal, and easy to fall. In terms of **fine motor**, she could draw a straight line, pull a zipper, unbutton buckles, cut paper inflexibly with scissors, eat with spoons and chopsticks, but couldn’t draw circles, squares, and triangles. In terms of the **language**, she could speak simple and long sentences with clear pronunciation, understand a few Chinese characters, recite numbers from 1 to 20, answer simple questions, but sometimes with confused word order and logic. For the **social adaptive capacity**, she could wear and take off clothes, shoes, and socks, could go to the bathroom, and eat by herself. She had the initiative to share and express her needs but without the initiative to say hello and goodbye. Besides, she had poor name calling response, poor sitting quietly ability, and hyperactive behavior.

The visually evoked potential (VEP) test showed that after blink flash stimulation for both eyes, N75, P100, and N145 waves could be induced with good repeatability. However, the latency of the P100 and N145 waves on both sides was prolonged, which was slightly abnormal. The evaluations for audiology system, heart, and urinary system were normal. DR X ray film for hip joint anteroposterior projection at 4-years-7-months old showed that the left and right acetabular angles were about 22 and 25°, respectively (Figure 2C). She was diagnosed with congenital dysplasia of the hip by an orthopedic surgeon at a tertiary children’s hospital. She received 2 brain MRI scans (March 28, 2019and October 26, 2021) and 3 electroencephalogram (EEG) examinations (March 15, 2019, August 20, 2020, and June 23, 2021). No obvious irregularities were identified.

Neuropsychological development assessment was performed for the patient at 5-years-1-month old using the Wechsler Preschool and Primary Scale of Intelligence Fourth Edition (WPPSI-IV) and the parent-rated Adaptive Behavior Assessment System II (ABAS-II) infant version. Her score on the full-scale intelligence quotient of WPPSI-IV was 75 (95% CI: 70-82, P5). The verbal comprehension index, visual spatial index, perceptual reasoning index, working memory index, and processing speed index of WPPSI-IV were 77 (95% CI: 71-86, P6), 83 (95% CI 76-94, P13), 79 (95% CI 73-87, P8), 76 (95% CI 76-94, P5), and 71 (95% CI 66-85, P3), respectively. The overall adaptive function score of ABAS-II was 77 (95% CI: 73-81, P6). The scores of social skills, conceptual skills, and practical skills in the three composite areas of adaptive function were 71 (95% CI 64-78, P3), 84 (95% CI 77-91, P14), and 80 (95% CI 74-86, P9), respectively. According to the clinical evaluation, she was at the edge level of intellectual development.

### Trio-WES Identified a *de Novo* Missense Variant of *ZMIZ1* Gene

Whole exome sequencing was performed for the trio to identify possible genetic factors of the proband. After removal of adaptors and low-quality reads, the obtained total clean data obtained for the trio were 11,959.04 (Mb) for the proband, 15,681.80 (Mb) for the father, and 12,202.51 (Mb) for the mother (Table 1). The target coverage was at least or more than 98%. The average depth of target region was more than 100X. The on-target ratio was more than 35%. In these samples, the total numbers of identified SNVs were 175,809 for the proband, 197,560 for the father, and 179,722 for the mother, respectively. The percentages of pathogenic variants were around 4%. The total number of small insertions and deletions were 36,452, 42,698, and 36,816 for the proband, father, and mother, respectively.

**TABLE 1:** Characterization of the whole exome sequencing for the trio.

Items	Proband	Father	Mother
Total clean data (Mb)	11,959.04	15,681.80	12,202.51
Target coverage	98.00%	98.31%	98.04%
Average depth of target region (X)	110.73	138.49	114.47
Ratio of average depth of target region (>4X)	97.59%	97.89%	97.64%
Ratio of average depth of target region (>10X)	97.29%	97.62%	97.36%
Ratio of average depth of target region (>20X)	96.77%	97.29%	96.85%
Ratio of average depth of target region (>30X)	95.64%	96.76%	95.71%
On target ratio	39.66%	37.83%	40.19%
Total SNVs	175,809	197,560	179,722
Percentage of pathogenic variants	3.57%	4.46%	3.47%
Total small insertion (and duplications)	17,011	19,694	17,254
Total small deletions	19,441	23,004	19,562

In this proband, 20 specific variants were selected. Twelve of them were heterozygous in 10 genes (*ANKRD36C, MUC2, MUC4, HRCT1, KLHL29, MYO15B, PER3, RPTN, TWIST1,* and *ZMIZ1*) and 8 homozygous in 8 genes (*AGAP3, CCDC177, CGN, DSPP, ESX1, FOXN4, MUC4, POTEB3,* and *SLC35E2A*) (Table 2). According to the criteria of ACMG guidelines, 10 heterozygous and 8 homozygous variants were annotated as variants of uncertain significance (VUS). Most of these variants were predicted to be “neutral” by Provean or “benign” by Polyphen. *PER3* (OMIM #603427), *TWIST1* (OMIM #601622), and *DSPP* (OMIM #125485) were also recorded in the OMIM database as disease-causing genes. However, the phenotypes caused by these genes were not in line with our female proband. Besides, two rare heterozygous variants (c.148C > T, p.R50W in *KLHL29* and c.2330G > A, p.G777E in *ZMIZ1*) were annotated as “likely pathogenic” (PS2 + PM2 + PP2) and “pathogenic” (PS2 + PM1 + PM2 + PP2 + PP3), respectively.

**TABLE 2:** Identified variants in the five-year-old proband.

No	Location (GRCH37)	Genes	Ref genes	Variants	dbSNP ID	Zygosity (P/F/M)	ACMG annotation	1000 genomes	ExAC	gnomAD exome	PROVEAN (score)	Polyphen2 (score)	Phenotype OMIM	Inheritance and phenotype
1	2:96521280	ANKRD36C	NM_001310154	c.5827A > C (p.I1943L)	rs112858216	Het/WT/WT	VUS: PS2	—	3.26E-02	—	Neutral −0.876	—	—	—
2	9:35906601	HRCT1	NM_001039792	c.317C > A (p.P106H)	rs112212538	Het/WT/WT	VUS: PS2	—	—	—	Neutral 0.071	Benign 0.146	—	—
3	2:23785214	KLHL29	NM_052920	c.148C > T (p.R50W)	rs558454968	Het/WT/WT	Likely pathogenic: PS2+PM2+PP2	—	1.52E-04	9.87E-05	Neutral −1.567	Damaging 0.988	—	—
4	11:1093349	MUC2	NM_002457	c.6863C > T (p.P2288L)	rs1382972456	Het/WT/WT	VUS: PM2	—	—	—	Neutral −0.461	—	—	—
5	3:195507271	MUC4	NM_018406	c.11180C > G (p.T3727S)	rs868067409	Het/WT/WT	VUS: PM2	—	1.67E-04	1.76E-04	Neutral 0.217	Benign 0.301	—	—
6	3:195508523	MUC4	NM_018406	c.9928G > A (p.A3310T)	rs879281830	Het/WT/WT	VUS: NA	—	2.79E-03	4.42E-04	Neutral 0.500	Damaging 0.494	—	—
7	3:195508526	MUC4	NM_018406	c.9925C > G (p.H3309D)	rs1424606542	Het/WT/WT	VUS: NA	—	2.43E-03	3.27E-04	Neutral 0.083	Benign 0.234	—	—
8	17:73585468	MYO15B	NM_001309242	c.1330C > T (p.R444C)	rs185791490	Het/WT/WT	VUS: PM2	—	—	—	Neutral −0.446	—	—	—
9	1:7890053	PER3	NM_016831	c.3019G > A (p.A1007T)	rs1776342	Het/WT/WT	VUS: PM2+PP3+BP4	—	—	9.47E-06	Neutral −0.848	Benign 0.004	#616882	AD: Advanced sleep phase syndrome, familial, 3
10	1:152129100	RPTN	NM_001122965	c.475G > A (p.G159S)	rs200003389	Het/WT/WT	VUS: PM2+BP4	—	—	—	Neutral −1.883	Benign 0.275	—	—
11	7:19156668	TWIST1	NM_000474	c.256_276dup (p.G86_G92dup)	—	Het/WT/WT	VUS: PS2+BP3	—	0	1.58E-05	—	—	#123100	AD: Craniosynostosis 1
													#180750	AD: Robinow-Sorauf syndrome
													#101400	AD: Saethre-Chotzen syndrome with or without eyelid anomalies
													#617746	AD: Sweeney-Cox syndrome
12	10:81064964	ZMIZ1	NM_020338	c.2330G > A (p.G777E)	—	Het/WT/WT	Pathogenic: PS2+PM1+PM2+PP2+PP3	—	—	—	Deleterious -7.736	Damaging 0.992	#618659	AD: Neurodevelopmental disorder with dysmorphic facies and distal skeletal anomalies (NEDDFSA)
13	7:150783920	AGAP3	NM_031946	c.92T > G (p.V31G)	rs1171186819	Hom/WT/WT	VUS: PM2	—	—	—	Neutral −0.091	—	—	—
14	14:70039807-70039809	CCDC177	NM_001271507	c.534_536del (p.A180del)	—	Hom/Het/Het	VUS: PM2+ PM3_supporting + BP3	—	—	—	—	—	—	—
15	1:151491411	CGN	NM_020770	c.416C > T (p.A139V)	rs181435993	Hom/Het/Het	VUS: NA	9.98E-04	7.44E-04	6.33E-04	Neutral −1.470	Damaging 0.937	—	—
16	4:88535832	DSPP	NM_014208	c.2018A > G (p.D673G)	rs201553143	Hom/Het/Het	VUS: PM2	—	1.98E-04	—	Neutral −1.162	Benign 0.004	#605594	AD: Deafness, autosomal dominant 39, with dentinogenesis
													#125420	AD: Dentin dysplasia, type II
													#125490	AD: Dentinogenesis imperfecta, Shields type II
													#125500	AD: Dentinogenesis imperfecta, Shields type III
17	X:103495090	ESX1	NM_153448	c.1040C > G (p.P347R)	rs200088361	Hom/Hemi/Het	VUS: NA	—	1.67E-03	1.04E-03	Neutral −0.236	Damaging 0.915	—	—
18	12:109719311	FOXN4	NM_213596	c.1195G > A (p.A399T)	rs146550988	Hom/Het/Het	VUS: NA	2.20E-03	1.35E-03	1.49E-03	Neutral −0.233	Benign 0.083	—	—
19	3:195506197	MUC4	NM_018406	c.12254A > G (p.D4085G)	rs148307810	Hom/Het/Hom	VUS: NA	7.39E-03	—	2.28E-03	Neutral −1.433	Damaging 0.553	—	—
20	15:22053725	POTEB3	NM_207355	c.1531A > G (p.K511E)	rs1949282	Hom/Het/WT	VUS: NA	—	—	—	Neutral 0.706	Benign 0.000	—	—

P, proband; F, father; M, mother; WT, wild type; Het, heterozygous; Hom, homozygous; Hemi, hemizygous; VUS, variants of uncertain significance; PS, strong pathogenic; PM, moderate pathogenic; PP, pathogenic supporting; BP, benign supporting; NA, not available; AD, autosomal dominant.

**FIGURE 3:**
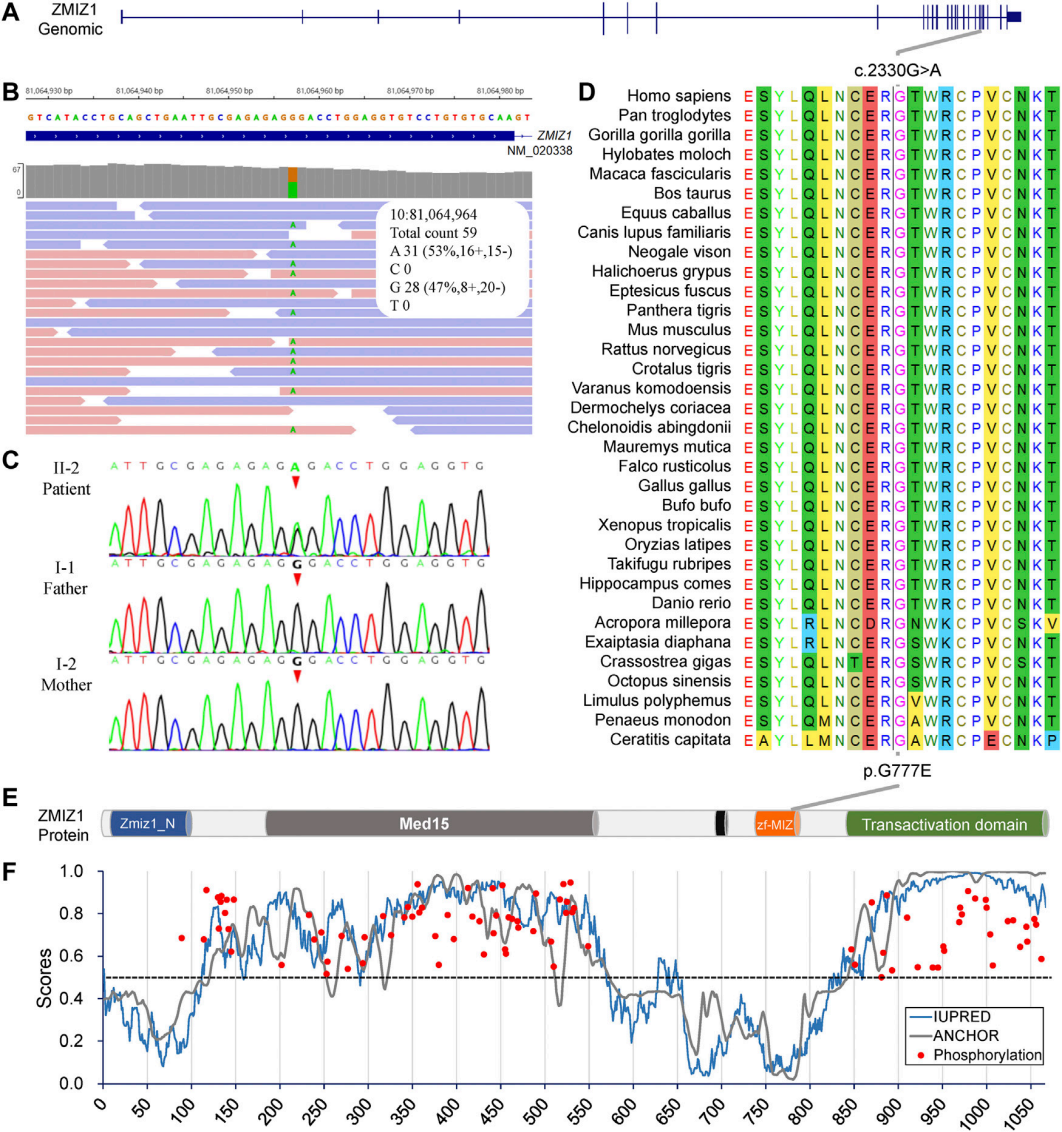
Molecular analysis of the c.2330G > A (p.G777E) in ZMIZ1 gene. **(A)** Gene structure of ZMIZ1; **(B)** IGV view of the c.2330G > A identified by WES; **(C)** Sanger sequencing of the c.2330G > A variant; **(D)** evolutionary conservation analysis; **(E)** protein structure of ZMIZ1; **(F)** analysis for intrinsically disordered regions and phosphorylation sites.

Since there was no experimental evidence for *KLHL29* leading to neurodevelopmental disorder, *ZMIZ1* was considered as the most potential disease-causing gene. The c.2330G > A (p.G777E) was a heterozygous SNV in the exon 20 of *ZMIZ1* gene (NM_020338) (Figures 3A,B) and confirmed by Sanger sequencing (Figure 3C) only in the patient, but not in her healthy parents or her elder sister. Thus, it was a *de novo* variant. The protein sequences of ZMIZ1 from more than 34 species (Mollusca, crabs, fish, amphibians, insects, reptiles, rodents, dogs, cats, cattle, and primates) were downloaded from NCBI GenBank and aligned by the ClustalW alignment algorithms of MEGA 11, the G777 was highly conserved in the animals during evolution (Figure 3D). G777E was localized in the functional MSX-interacting zinc finger (zf-MIZ) domain (Figure 3E) and predicted to be “deleterious” with a score of 0.536, “deleterious” with a score of -7.736 (Provean) and “probably damaging” with a score of 0.992 (PolyPhen-2). In addition, this SNV has not been detected in multiple public genome databases, such as 1000 Genome Project (*n* = 2504), NHLBI Exome Sequencing Project (GO-ESP) (*n* = 6503), the Exome Aggregation Consortium (ExAC) (*n* = 60,706), Genome Aggregation database (gnomAD) (*n* = 15,708), and NHLBI Trans-Omics for Precision Medicine (TOPMED) (*n* = 60,000). Since this amino acid changing variant was only identified in the patient, and not in her parents, it was regarded as “spontaneous.” According to the Probability of Loss-of-function Intolerance (pLI) analysis, the pLI value of ZMIZ1 was 1.000, which indicated *ZMIZ1* being a haploinsufficient gene. It has been reported that *ZMIZ1* could cause the occurrence of a rare neurodevelopmental disorder, neurodevelopmental disorder with dysmorphic facies and distal skeletal anomalies (NEDDFSA). Based on the recorded clinical phenotypes (Supplementary Table S2), this patient was finally diagnosed as NEDDFSA. Besides, four rare variants in *ZMIZ1* were also detected in another four NEDD patients, namely, c.3096 + 15C > T, c.1024A > G (p.M342V), c.540 + 20T > C and c.679G > A (p.A227T) (Supplementary Table S3).

The intrinsically disordered regions of ZMIZ1 protein (NP_065071) were analyzed using the online web server IUPred2A. Two functional globular regions were identified at two portions (aa2-110 and 575-820), which overlapped with the two important functional domains, Zmiz1 N-terminal tetratricopeptide repeat domain (Zmiz1_N, aa8-100) and MIZ/SP-RING zinc finger (zf-MIZ, aa739-786), respectively. The predicted two globular regions and two intrinsically disordered regions are displayed in Figure 3E. The possible phosphorylation sites of ZMIZ1 were predicted using DEEP under default parameters. Interestingly, most of the phosphorylation sites were located in the two long disordered regions (Figure 3F). As for G777E, it was localized in the second globular regions containing zf-MIZ domain and might affect the probable tertiary structures as predicted by I-TASSER (Supplementary Figure S1).

### Analysis of the Distinct Regions of ZMIZ1

**FIGURE 4:**
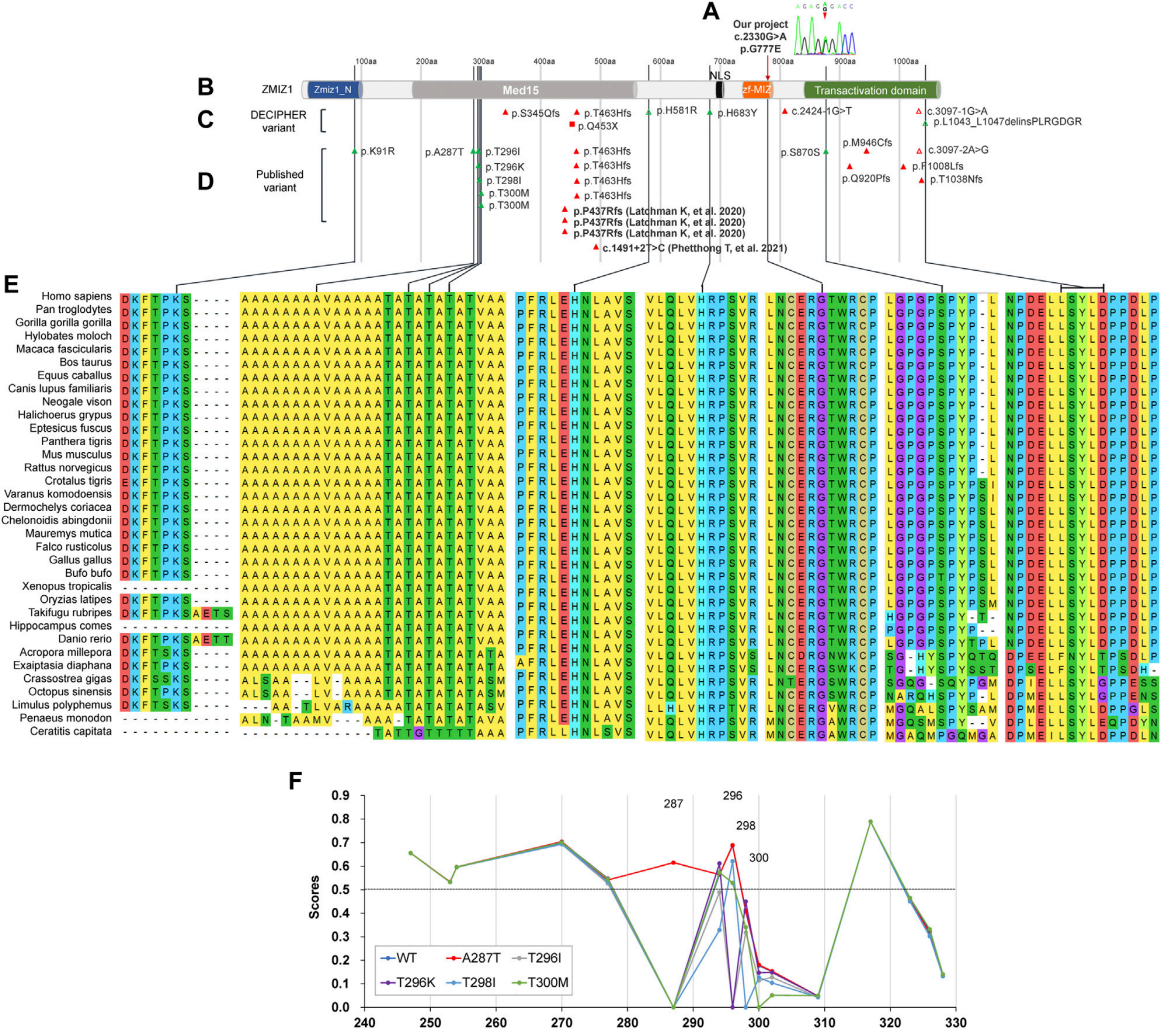
Molecular analysis of variants of ZMIZ1. **(A)** Variant in our cohort; **(B)** diagram of ZMIZ1 protein; **(C)** variants in DECIPHER; **(D)** variants in reported articles; **(E)** evolutionary conservation; **(F)** phosphorylation analysis in the alanine-rich region.

The distinct regions of ZMIZ1 were analyzed using the online software Motif Scan under default settings. Seven distinct regions were identified, one alanine-rich region (aa280-305, E-score = 2.1E-06), two proline-rich regions (aa334-555, E-score = 3.9E-16; aa867-1002, E-score = 3.8E-07), one bipartite nuclear localization signal (NLS, aa697-711, E-score = 2.1E+04), MIZ/SP-RING zinc finger (aa738-787, E-score = 1.1E-33), and one copper binding octapeptide (aa947-954, E-value = 1.5). All variants of ZMIZ1 were also recruited from the DECIPHER database (Swaminathan et al., 2012) and the four published articles (Córdova-Fletes et al., 2015; Carapito et al., 2019; Latchman et al., 2020; Phetthong et al., 2021). A total of 33 patients with ZMIZ1 pathogenic variants were collected, 1 from our current cohort (Figures 4A,B), 8 from the DECIPHER database (Figure 4C), and 24 from published articles (Figure 4D). Except for K91R, H581R, and H683Y, other variants were localized in the low-complexity regions, such as the alanine-rich region and the proline-rich region of the Med15 (mediator complex subunit 15) domain, and the proline-rich region in the C-terminal transactivation domain (TAD). There were nine amino acid-changing variants, which were strongly conserved during evolution (Figure 4E). Six of them were in the alanine-rich region, accounting for 66.67% (6/9). From the phosphorylation prediction by DEEP, except for T300M, other variants could distinctly change the phosphorylation pattern of the alanine-rich region (Figure 4F).

### Interaction Network of ZMIZ1

**FIGURE 5:**
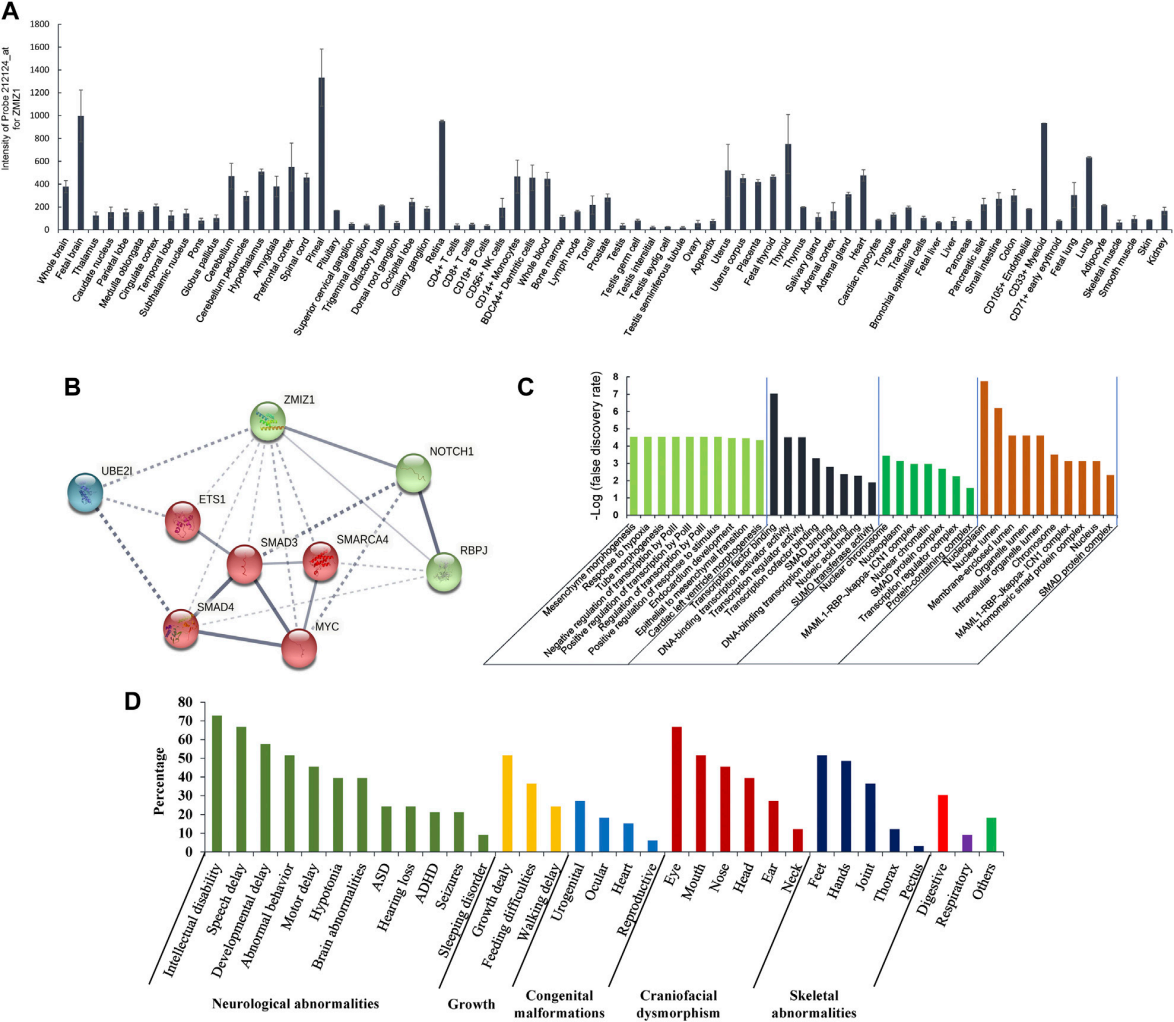
Expression of ZMIZ1 in different human tissues, and network analysis. **(A)** Tissue-specific expression of ZMIZ1; **(B)** protein interaction network produced by STRING; **(C)** GO analysis for the 9 members of the network; **(D)** phenotypes of the patients carrying ZMIZ1pathogenic variants.

The gene expression data of 79 human tissues showed that *ZMIZ1* was expressed in the heart, thyroid, immune cells, ovary, retina, and brain, with the highest in the pineal (Figure 5A). The protein interaction network with ZMIZ1 indicated that ZMIZ1 could interact with SMAD3, SMAD4, MYC, NOTCH1, RBPJ, SMARCA4, ETS1, and UBE2I (Figure 5B). According to the GO analysis for the 9 members (Figure 5C), the network was involved significantly in mesenchyme morphogenesis, hypoxia, tube morphogenesis, regulation of transcription, response to stimulus, endocardium development, epithelial to mesenchymal transition, and cardiac left ventricle morphogenesis in GO term “biological process.” In “molecular function,” transcription, SMAD binding, and SUMOylation were significantly enriched. As for “cellular component” and “subcellular localization,” members of this network were localized in nuclear to form multiple protein complexes, mainly MAML1-RBP-Jκ-ICN1 (Intracellular Notch1) complex and SMAD protein complex to regulate the expression of target genes.

## Discussion

Pathogenic variants of the zinc finger MIZ-type containing 1 (*ZMIZ1*, OMIM#607159) could cause the occurrence of a rare syndromic disease, neurodevelopmental disorder with dysmorphic facies and distal skeletal anomalies (NEDDFSA) with an autosomal dominant (AD) mode of inheritance. Currently, 32 patients with neurodevelopmental disorders have reported carrying pathogenic variants in the protein coding sequences of *ZMIZ1* (*n* = 29) and chromosomal translocations involving ZMIZ1 (*n* = 3) (Supplementary File S2). Among these patients, except for c.1491+2T > C identified in a Thai female, the remaining 31 variants were detected in patients with Caucasian origin in Western countries. In our small cohort of NEDD/ID cases in China, a *de novo* missense pathogenic variant c.2330G > A (p.G777E) was detected in a 5-year-old girl. This patient presented the characteristic clinical phenotypes of NEDDFSA, such as neurodevelopmental delay, mild intellectual disability, hypotonia, language delay, dysmorphic facial features, joint hypermobility, and hand and foot anomalies, which were the common features of NEDDFSA (Figure 5D, Supplementary Table S2). As far as we know, this was the first report of *ZMIZ1* variant in Chinese. Besides, we also identified four other rare variants in the ZMIZ1 gene (c.540 + 20T > C, c.679G > A, c.1024A > G, and c.3096 + 15C > T) (Supplementary Table S3). Although predicted as “neutral” or “benign” to the function of ZMIZ1, it still could not rule out their pathogenicity. Cellular and animal experiments should be taken to verify the function of these variants, including the c.2330G > A (p.G777E).

*ZMIZ1* was previously known as ZIMP10, RAI17, or KIAA1224. In 1999, Nagase et al. identified the gene *ZMIZ1* (previously called KIAA1224) from a fetal brain cDNA library (Nagase et al., 1999). According to the human tissue-specific transcriptomes, it was expressed in the heart, thyroid, immune cells, ovary, retina, and brain, with the highest in the pineal gland (Su et al., 2004). The encoded protein is a transcriptional co-activator, which belongs to the Protein Inhibitor of Activated STAT (PIAS) family. As a member of the PIAS family, ZMIZ1 has a highly conserved MIZ (Msx-interacting zinc finger) domain which is important for protein-protein interaction and SUMOylation (Sharma et al., 2003; Beliakoff and Sun, 2006). It had been reported that ZMIZ1 could regulate the activity of many transcription factors, such as androgen receptor (AR) (Beliakoff and Sun, 2006), SMAD3 (Li et al., 2006), SMAD4 (Li et al., 2006), and p53 (Lee et al., 2007). As an ortholog of ZMIZ1, tonalli (tna) was identified in *Drosophila melanogaster* and interacted with the ATP-dependent SWI/SNF complexes, which suggested a potential role in chromatin remodeling (Gutiérrez et al., 2003). Recently, ZMIZ1 was identified to be interacted with BRG1 (SMARCA4) (Li et al., 2011), BAF57 (SMARCE1) (Li et al., 2011), or SATB1 (Pinnell et al., 2015) to regulate the chromatin remodeling in humans. The protein-protein interaction network showed that ZMIZ1 could interact with SMAD3, SMAD4, MYC, NOTCH1, RBPJ, SMARCA4, ETS1, and UBE2I. According to the GO analysis for the 9 members of the protein network containing ZMIZ1, the network was significantly involved in mesenchyme morphogenesis, hypoxia, tube morphogenesis, regulation of transcription, response to stimulus, endocardium development, epithelial to mesenchymal transition, and cardiac left ventricle morphogenesis. This explained why *ZMIZ1* pathogenic variant could affect the normal development of multiple systems, such as nerve, heart, and bones. GO analysis also showed that members of this network were localized in the nucleus to form two multiple protein complexes, mainly MAML1-RBP-Jκ-ICN1 complex and SMAD protein complex, to regulate the expression of target genes. The proper expression of *ZMIZ1* was essential for the standard embryonic development. It has been revealed in mice embryos at different stages that *ZMIZ1* was expressed dynamically in the neural tissues, craniofacial tissues, mandibular, foregut, limb buds, optic vesicle and otic pit, and somite (Beliakoff et al., 2008; Rodriguez-Magadán et al., 2008). This was consistent with the above-mentioned clinical features produced by the mutant ZMIZ1.

After compiling all the *ZMIZ1* variants in the DECIPHER database, published articles, and our cohort (Supplementary Table S2), 12 patients were found to carry amino-acid changing variants, and half of them (6/12) had variants in the alanine-rich sequence. The alanine-rich low-complexity region (LCR) was localized in the N-terminal intrinsic disordered region of ZMIZ1. The alanine-rich sequences were extremely conserved in different species during evolution, suggesting its importance for the proper function of ZMIZ1. According to the reports, many transcription factors or transcription mediators, such as FUS (FUS RNA binding protein), EWSR1 (EWS RNA binding protein 1), TAF15 (TATA-box binding protein associated factor 15), Sp1 (Sp1 transcription factor), and AR could interact with ZMIZ1 at the transcriptional start sites via their extremely low-complexity regions (LCRs) to form local phase-separated condensates (or called droplets) to stabilize DNA binding, recruit RNA polymerase II (RNA Pol II), and activate transcription (Chong et al., 2018; Zamudio et al., 2019). These special condensates were a trade-off between proper functionality and risk of abnormal aggregation. The aberrant phase transitions within liquid-like droplets lie at the heart of many kinds of diseases, such as TATA box-binding protein (TBP, OMIM#600075) for spinocerebellar ataxia 17 (SCA17, OMIM#607136) (Friedman et al., 2007), FUS (OMIM#137070) for amyotrophic lateral sclerosis 6 (ALS6, OMIM#608030) (Patel et al., 2015), and androgen receptor (AR, OMIM#313700) for spinal and bulbar muscular atrophy (SBMA, OMIM#313200). As predicted by IUPred2A, ZMIZ1 contained three low-complexity regions (one alanine-rich and two proline-rich regions). It is reasonable that the alanine-rich region might be indispensable for the phase separation of ZMIZ1 to carry out the transcription mediator function. As predicted, the variants could change the phosphorylation pattern in the alanine-rich region, which might affect the local conformation. This might be the underlying molecular mechanism for the alanine-rich region being the variation hotspot of ZMIZ1. However, this has not yet been experimentally verified.

## Conclusion

In conclusion, a *de novo* missense variant was first discovered in a Chinese female with a rare heterozygous syndromic disease, neurodevelopmental disorder with dysmorphic facies, and distal skeletal anomalies (NEDDFSA). Currently, a total of 32 patients with 27 types of variants of *ZMIZ1* (24 in protein-coding sequences and 3 translocations) have been identified globally. However, the underlying molecular mechanism of these variants has not been elucidated. Further experimental studies should be carried out to clarify these unknown fields to determine potential drug targets for the treatment of NEDDFSA.

## Data Availability

The datasets for this article are not publicly available due to concerns regarding participant/patient anonymity. Requests to access the datasets should be directed to the corresponding authors.
