# Supplementary material for: A de Novo ZMIZ1 Pathogenic Variant for Neurodevelopmental Disorder With Dysmorphic Facies and Distal Skeletal Anomalies
Source: Front Genet. 2022 Mar 31;13:840577. doi: 10.3389/fgene.2022.840577 (PMC9008544; doi:10.3389/fgene.2022.840577)
Supplement: Supplementary file 4 [file Image1.pdf]

Supplementary figure 1

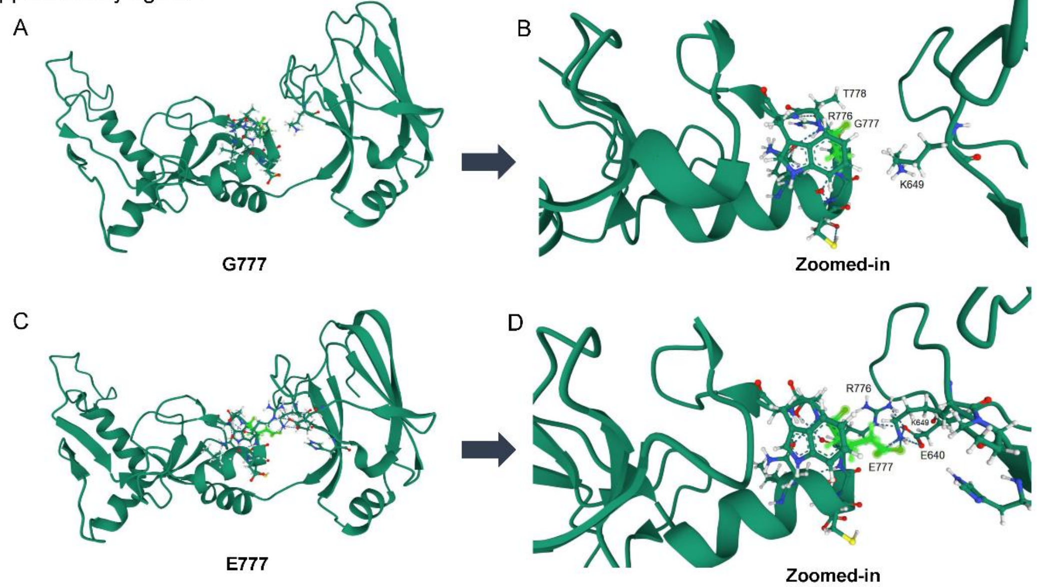

Supplementary figure 1. Structural prediction for ZMIZ1. A) Structure prediction of the sequence containing G777 (575-820) by I-TASSER; B) Zoomed-in view of structure containing G777; C) Structure prediction of the sequence containing E777 (575-820); D) Zoomed-in view of structure containing E777.

The effect of G777E on the probable tertiary structures were predicted by I-TASSER under default parameters for the whole second globular region (aa575-820). As predicted, the amino acid sequences around G777 formed a helix (766-776) and a coiled-coil (777-789), G777 interacted with N773 to stabilize the helix structure and keep the hydrophilic guanidine group of R776 inside the helix to interact with T778 via hydrogen bonds (Supplementary figure 1-A, -B). However, E777 releases R776 from the helix. They interacted with K649 and E640, respectively. The K649 and E640 were also bound with each other (Supplementary figure 1-C, -D). Thus, these four amino acids intertwined to form a closed spatial constructure, which might be deleterious for zf-MIZ domain to interaction with its partners.
